# Universal Adhesive for Fixed Retainer Bonding: In Vitro Evaluation and Randomized Clinical Trial

**DOI:** 10.3390/ma14061341

**Published:** 2021-03-10

**Authors:** Maria Francesca Sfondrini, Simone Gallo, Benedetta Turcato, Mona A. Montasser, Nehal Fouad Albelasy, Pekka K. Vallittu, Paola Gandini, Andrea Scribante

**Affiliations:** 1 Unit of Orthodontics and Paediatric Dentistry, Section of Dentistry, Department of Clinical, Surgical, Diagnostic and Paediatric Sciences, University of Pavia, 27100 Pavia, Italy; francesca.sfondrini@unipv.it (M.F.S.); benedetta.turcato01@universitadipavia.it (B.T.); paola.gandini@unipv.it (P.G.); 2 Orthodontic Department, Faculty of Dentistry, Mansoura University, Mansoura 35516, Egypt; mmontasser11@mans.edu.eg (M.A.M.); nelbelasy@mans.edu.eg (N.F.A.); 3 Institute of Dentistry, Department of Biomaterials Science and Turku Clinical Biomaterials Centre, University of Turku and City of Turku, Welfare Division, 20520 Turku, Finland; pekka.vallittu@utu.fi

**Keywords:** adhesion, bonding, clinical trial, mandibular, maxillary, multistranded wire, orthodontics, fixed retainer, splint, universal adhesive

## Abstract

This study aims to assess the efficacy of a universal adhesive (Scotchbond Universal, 3M ESPE) (SB) in total-etch mode, compared to a traditional orthodontic primer (Transbond XT Primer, 3M ESPE) (XT Primer), to perform bonding of orthodontic fixed retainers along with the Transbond XT Light Cure Adhesive Paste (3M ESPE). For the in vitro study, a round section wire (Ortosmail Krugg) was bonded using XT Primer for 20 bovine incisors (Group 1) and SB for other 20 (Group 2). Samples were debonded in a universal testing machine applying a tangential force to specimens (crosshead speed of 1 millimeter per minute). Shear bond strength (SBS) and adhesive remnant index (ARI) scores were calculated. For the in vivo study, 100 patients needing upper and lower canine-to-canine fixed retainers after orthodontic treatment were randomly assigned to two groups of 50 participants each, i.e., group 1 (retainer bonding with XT Primer) and group 2 (retainer bonding with SB). Over two years, examinations were carried out monthly, and detachments were registered by considering the teeth and arches affected. In vitro, no statistically significant differences in SBS and ARI scores were demonstrated between the two groups, both showing a mean bond strength of about 12 MPa and major frequency of ARI “2” (>50% remnant adhesive on the enamel). Conversely, a significantly lower failure rate over 2 years was assessed clinically for group 2 in both arches. Independently of the adhesive and arch, incisors reported a significantly higher failure rate than canines. Scotchbond Universal used in total-etch mode could be a valid alternative to the traditional orthodontic Transbond XT Primer.

## 1. Introduction

After obtaining the desired position of the teeth, the final step of an orthodontic therapy consists of a retention procedure that attempts to keep the result achieved, avoiding a certain relapse due to the tendency of teeth to regain their pretreatment position [1]. This phenomenon, occurring especially after rotation movements, has been considered the consequence of the supracrestal fibers’ stretching, whereas other authors suggest that the elastic properties of the whole gingival tissue are responsible for this [2,3]. At present, one of the most common strategies consists of a prolonged retention period with a bonded metal [4] or a fiber-reinforced composite [5] fixed retainer. These retention methods are usually preferred for the mandibular arch but also used for the maxillary one [6].

Despite this appliance not necessitating the active cooperation of the patient as required for removable retainers, its main disadvantage remains the risk of detachment [7,8]. The causes of this event are represented by the use of too little adhesive or distortions during its setting, direct trauma to the retainer, low resistance to the fatigue, or unfavorable occlusal contacts with abrasion of the composite [9]. In order to guarantee a long-lasting retention of the wire to teeth, proper characteristics of the adhesive are required, including an adequate adhesion force and wire pull-out resistance, along with hardness, wear resistance, low water absorption, and low vulnerability to aging mechanisms [10]. On the other hand, due to the need of removing the orthodontic device once the treatment outcome is obtained, the bonding system should not provide too high bond strength because removal of the device can cause damage to the enamel [11].

Different adhesive systems have been developed in the restorative field. Currently, the most recent generation is known as “universal” or “multimode”, and the key point of these adhesives consists of the possibility of being used either with the total-etch mode or with the self-etch one, according to the choice of the operator [12]. In any case, the bond strength of these materials to enamel has been shown to be significantly improved with the first method, which consists of a pre-etching with orthophosphoric acid performed on the enamel before their application [12,13,14].

Until now, several studies have evaluated which composite resin is associated with the lowest incidence of retainers’ failure: Conflicting results have been obtained, as some authors found no statistically significant differences between the forces required to detach the different samples [15,16], whereas others suggested that orthodontic resin should be considered the most reliable material [17,18]. However, in these abovementioned in vitro studies, exclusively orthodontic adhesive systems were being compared to each other. Conversely, to the best of our knowledge, the efficacy of a traditional orthodontic primer has never been compared to a universal adhesive (applied in total-etch mode) that is generally used in restorative dentistry. As previously stated, many authors in the restorative field have shown that the bonding force of a universal adhesive greatly increases if enamel etching is performed before the product application [19,20,21,22,23,24,25]. In particular, the studies of Beltrami et al. [13], de Goes et al. [14], and Suzuki et al. [26] compared different universal adhesives by evaluating the increase in detachment forces with and without enamel pre-etching. All studies show how this procedure led to a significant increase in the bonding force values of the tested universal adhesives. Therefore, it has been demonstrated that the application of a universal adhesive with the total-etch mode has a positive effect on the durability of the bond to the enamel in restorative dentistry. Specifically, Schotchbond Universal includes into its chemical composition the 10-Methacryloyloxydecyl dihydrogen phosphate (10-MDP) monomer, which can establish a chemical bond to dental substrates. The functional monomer has a proven ability to chemically interact with the hydroxyapatite forming stable MDP-Ca salts; since universal adhesive systems tend to have higher pH values compared to the orthophosphoric acid used in the total-etch adhesion, which decreases their capability to etch the enamel, a pre-etching phase of this latter before the application of the adhesive is strongly recommended [27].

On the basis of this consideration, it may be hypothesized that the usage of the universal restorative adhesive following the enamel pre-etching could represent a reliable option to avoid retainers’ detachments. Accordingly, the aim of this current in vitro and in vivo study is to analyze even in orthodontics the efficacy of a universal adhesive generally used in restorative dentistry, which is applied here after enamel pre-etching in order to bond fixed retainers to teeth. The research question is whether this last bonding agent is found to be more effective in orthodontic adhesion with respect to a conventional orthodontic primer.

The first two null hypotheses investigated in vitro are that there is no significant difference in shear bond strength (SBS) and in the adhesive remnant index (ARI) scores between the two different products tested when used with the same adhesive paste. The third and fourth null hypotheses investigated through a randomized clinical trial are that during a 2-year follow up there are no significant differences in the fixed retainers’ bond failure rates and that there are no significant differences between incisors and canines failure rate.

## 2. Materials and Methods

### 2.1. In Vitro Experimentation

This experiment has been approved by the Unit Internal Review Board. Specimens were prepared from bovine incisors recently extracted. Considering the difficulty in obtaining human teeth for laboratory studies, the bovine ones represent a valid substitute, considering the similarity of the enamel [28].

After the extraction, teeth were kept in complete darkness in a solution of 0.1% (wt/vol) thymol without alcohol [29]. Within seven days from the extraction, soft tissues debris were removed using a scalpel, and teeth were cleaned with a toothbrush, washed, and dipped into a renewed thymol solution. Subsequently, each one was carefully examined at ×10 magnification with the use of a microscope (Stereomicroscope SR, Zeiss, Oberkochen, Germany) and was excluded in case of fractures, caries and alterations of the labial enamel.

Sample size calculation (alpha = 0.05; power = 95%) was performed considering two independent study groups (Transbond XT Primer and Schotchbond Universal) and a continuous variable. Concerning the primary outcome (bond strength), an expected mean of 24.7 was hypothesized, with a standard deviation of 9.2 [30,31]. The expected difference between the means was supposed to be 10.5, and therefore 20 specimens were requested for each group. A total sample size of 40 teeth was finally selected according to the sample size calculation.

The root of each element was embedded into cold-curing fast-setting acrylic (Leocryl, Leone s.p.a., Sesto Fiorentino, Italy) inside a plastic cylindrical mold (2 cm height × 2 cm diameter). Teeth were positioned in the center of the molds and with an inclination, thus allowing a shear force to act on their labial surface.

The 37% orthophosphoric acid (Gerhò Etchant gel 37%, Gerhò spa, Terlano, Italy) was used to etch buccal enamel for 30 seconds, as generally recommended in dentistry [32], and then teeth were rinsed thoroughly for other 30 seconds. Subsequently, an area of 3 mm^2^, calculated by delimiting with a pencil the presence of a 3 mm × 1 mm retainer, was immediately bonded.

For this phase, samples were randomly divided into two groups of 20 specimens each. In group 1 (control, XT Primer), the bonding agent used was Transbond XT Light Cure Adhesive Primer (3M ESPE, Saint Paul, MN, USA) whereas in group 2 (trial, SB) Scotchbond Universal (3M ESPE, Saint Paul, MN, USA) was used.

The assigned bonding material was applied by means of a brush, following the specific procedures listed in Table 1 and per manufacturer’s instructions.

On each sample, a multistranded round section wire (Ortosmail, Krugg, Milan, Italy), of 3 mm length, was applied along with Transbond XT Light Cure Adhesive Paste (3M ESPE) whose excess was removed with a probe to limit an area of 3 mm^2^. In conclusion, light curing of the paste was performed for 10 seconds in an occlusal–apical direction and for 10 seconds in the contrary one.

The LED lamp Starlight Pro (Mectron s.p.a., Carasco, Italy), with an output irradiance of 1000 mW/cm^2^, was used when necessary for all the light curing phases. The distal end of the light guide was placed perpendicular and at contact with the resin to be cured.

The characteristics of the tested materials and the protocols recommended for their application are shown in Table 1.

All the specimens were then stored in distilled water for 24 h at room temperature before proceeding with the subsequent experimental phase.

#### 2.1.1. Shear Bond Strength (SBS) Test

**Figure 1:**
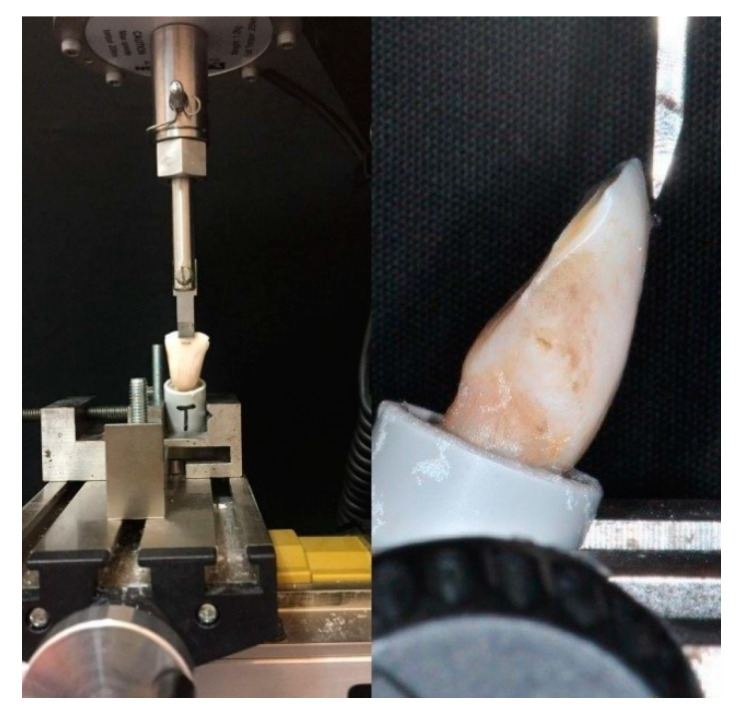
Positioning of samples into the Instron machine: (**left**) frontal view; (**right**) lateral view.

Each specimen was placed in a universal testing machine (Model 3343, Instron, Canton, MA, USA). According to a previous study of our group [30], retainers were exposed to a force exerted by a steel tip in an occlusogingival direction, tangentially to the adhesion surface, as far as the breakdown of bonding, as exhibited in Figure 1. The crosshead speed was configured to 1 millimeter per minute [33]. Teeth were secured in the lower jaw of the machine so that the retainer’s long axis was parallel to the edge of the blade. The loading test was performed dry at room temperature.

The software Bluehill 2 (Instron Industrial Products, Grove City, Pennsylvania, PA, USA) automatically recorded the maximum detachment shear force in Newtons. Results were then transformed into megapascal, with the following formula:

Shear force (MPa) = N/mm^2^ (1)

#### 2.1.2. Adhesive Remnant Index (ARI) Score

After detachment, retainers and teeth surfaces were analyzed under a ×10 optical microscope (Stereomicroscope SR; Zeiss, Oberkochen, Germany) [34], and the amount of adhesive remaining on each tooth surface (primer/enamel interface) was measured according to the adhesive remnant index (ARI) score (0: 0% remnant adhesive; 1: ≤50% remnant adhesive; 2: >50% remnant adhesive; and 3: 100% adhesive remnant adhesive) to disclose the amount of adhesive present after the detachment, as reported by previous studies [30,35].

### 2.2. In Vivo Study

- Trial design

This study was conceived as a parallel-group, active controlled, and single-center randomized clinical trial (RCT) with a 1:1 allocation ratio. This study has been approved by the Unit Internal Review Board Committee (2016 0113). No variations to the methods occurred during the study. In order to reduce bias, blinding was conceived for patients, the outcome assessor, and data analyst.

- Participants

Patients referred to the affiliation of the authors were engaged from February 2016 to January 2018 and were followed until January 2020. For each patient, the informed consent was achieved (or the parents’ one on behalf of underage patients). Interventions and data assessment were carried out in the same center.

**Figure 2:**
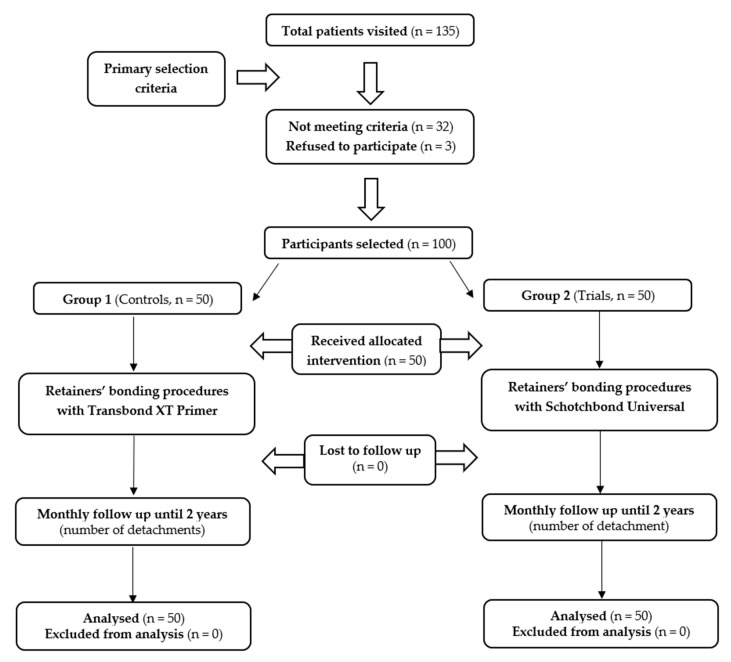
Flow chart showing participants and the protocol used in this study.

The inclusion criteria were going towards the final step of an orthodontic treatment and the later positioning of both upper and lower fixed retainers. Patients reporting facial trauma, onychophagia, and habitude of biting pencils or pens were excluded from this study. Among the participants selected, three refused to participate. The flow of the participants and the investigation protocol used in the present report are showed in Figure 2.

- Interventions

At the end of the orthodontic treatment, participants were randomly subdivided into two groups, in accordance with the product later used for the retainer’s bonding. The materials tested are those previously reported for the in vitro evaluation. Multistranded wires were bonded on upper and lower arches on the palatal/lingual side, involving incisors and canines. In the first group (XT Primer), the adhesion procedure was conducted using the conventional Transbond XT Primer (3M, St. Paul, MN, USA) and in the second group (SB), the universal adhesive Scotchbond Universal (3M). All the other steps, e.g., the use of the Transbond XT Light Cure Adhesive Paste, were the same in the two groups. To guarantee a standardization, interventions were always realized by a same operator who was not involved in the previous in vitro study.

- Outcomes

From the date of retainer’s bonding, participants of both groups underwent a 2-year follow up, with periodic visits every month, executed by another operator not taking part in the bonding phase or the in vitro study. Each detachment of retainers was recorded, considering the position (maxillary or mandibular arch) and the respective number of incisors and canines involved. Only the first temporal detachment (or detachments) was been considered, whereas subsequent ones were not further contemplated for the statistics. Participants were instructed to respect the planned appointments and to urgently contact the dentist in case of any suspected detachment.

- Sample size

The established alpha was 0.05 and power was 80%, and considering two independent groups and a continuous primary outcome, the sample size calculation required 100 total participants resulting in 1200 teeth splinted. Concerning the primary outcome (failure incidence), an expected frequency of 30% was hypothesized. The expected difference between the frequencies was supposed to be 21.8. This margin was supposed by basing on the results of previous reports that evaluated similar topics [36,37].

A total of 50 patients were requested for each group. Loss to follow-up and incomplete compliance with therapy were excluded. A total sample of 100 patients was finally selected of which 48.5% males (mean age: 25 years and 9 months) and 51.5% females (mean age: 25 years and 3 months). Among the 50 controls, 49% were males (mean age: 27 years and 2 months) and 51% females (mean age: 26 years and 11 months), whereas, among the 50 trials, 48% were males (mean age: 24 years and 2 months) and 52% females (mean age: 23 years and 10 months).

- Randomization and blinding

Randomization sequence was performed by the data analyst using a block randomization table and considering a permuted block randomization with 50 participants for each fixed block. Participants were enrolled by the dental operator who also achieved the allocation concealment by using sequentially numbered and sealed envelopes containing the allocation cards earlier arranged. It has not been technically feasible to blind the operator. On the contrary, participants, the outcome detector, and statistician were blinded throughout the whole trial since they were not aware of the treatment assigned. No visible significant differences could be noticed during the data assessment between the retainers bonded with the two different methods.

### 2.3. Statistical Analysis

The results achieved were analyzed using the R Software (R version 3.1.3, R Development Core Team, R Foundation for Statistical Computing, Wien, Austria). The statistical significance for all the analyses was set at *p* < 0.05.

As regards the shear bond strength values, descriptive statistics (mean, standard deviation, minimum, median, and maximum values for each group) were calculated. Data normality was determined through the Kolmogorov–Smirnov test. Homoscedasticity was verified using Levene’s test. The *t*-test was applied to assess the presence of significant differences in debonding values between the two groups. To reveal significant differences in adhesive remnant index scores, a frequency analysis represented by an χ^2^ test was carried out.

The Fisher exact test was applied to assess differences in the frequencies of the clinical detachments of the groups tested in vivo; moreover, the relative Kaplan–Meier survival curves of the adhesives examined were designed and compared by means of the log-rank test.

## 3. Results

### 3.1. In Vitro Experimentation

#### 3.1.1. Shear Bond Strength (SBS) Test

As reported in Table 2, a *t*-test demonstrated the absence of a significant difference between the two groups (*p >* 0.05), despite a major SBS assessed for group 2.

#### 3.1.2. Adhesive Remnant Index (ARI) Score

The χ^2^ test (value = 2.067) did not show any significant difference between the two groups (*p* > 0.05), which both exhibited a higher prevalence of ARI scores “2”, as reported in Table 3.

### 3.2. In Vivo Study

At the end of the follow up, statistically significant differences were found in terms of detachments (failure rate) between the two groups of the study (*p* < 0.05). Considering both arches as well as the upper and lower ones separately, the trial group (group 2) reported significantly lower total detachments. Within group 1, lower teeth had a significantly higher failure percentage (*p* < 0.05) with respect to the upper ones (27.33% vs. 16.33%), and conversely, within group 2, a higher failure rate was seen for the upper teeth (7.33% vs. 6.00%) but without a statistical significance (*p >* 0.05).

Moreover, when the bonding performance was compared between the two different kinds of teeth, the percentages of splint failures were significantly higher for incisors with respect to canines, considering both groups independent of the arch (Table 4). No bone strains were noticed.

**Figure 3:**
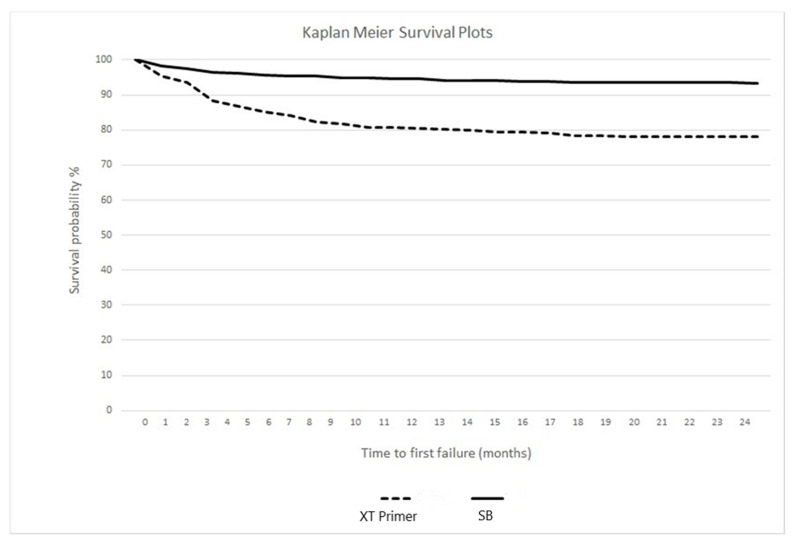
Kaplan–Meier survival curves for the adhesive systems in the two groups.

Kaplan–Meier survival curves are showed in Figure 3. A statistically major failure risk over the 24 months of follow up was assessed for group 1 (hazard ratio: 3.77; 95% confidence interval: 2.46–4.52; log rank test: *p* < 0.00001). During the 2-year retention period no wire fractures were found in both groups.

## 4. Discussion

The first two null hypotheses of the present study were accepted. The results obtained in vitro showed the absence of statistically significant differences between the conventional orthodontic primer and the universal adhesive, for both SBS and ARI. As regards this latter parameter, a major frequency of an ARI score of 2 has been shown for both materials tested, which indicates that a cohesive failure at the wire–composite interface occurs, and more than 50% of the adhesive remains on the teeth, with no failure of the bonding interface (enamel–adhesive), in accordance with the results reported by other authors [38,39]. Considering that a good orthodontic biomaterial should report an ARI score of 1 or 2 and that it should have a bonding strength in the interval of 5–50 MPa to sustain masticatory forces [40], the values of the two respective tested materials fall within these ideal ranges, but no significant difference was detected between them.

According to these results, both Scotchbond Universal used in the total-etch mode as well as the conventional orthodontic primer appear to be adequate systems since they showed no bonding failure at the enamel–adhesive interface but only detachments at the composite–wire site (cohesive fracture). To date, we found no studies in literature comparing the average force required for the detachment of splints bonded with Transbond XT Primer and Scotchbond Universal. This prevents us from directly comparing these results with those of analog studies. Transbond XT Primer has been chosen as the only reference of non-universal adhesives because of its wide use in orthodontic bonding.

A limitation of this in vitro study might be represented by the fact that it was conducted on bovine teeth and not on human ones, due to the difficulty in obtaining the latter for research purposes. As stated in the systematic review with a meta-analysis conducted by Soares et al., [41] despite some studies pointing out certain differences between the two types of teeth that could impact on bonding performance, the use of bovine teeth in bond strength tests produces comparable results to human ones, both on enamel and dentin. Accordingly, bovine teeth can be considered as reliable tools for the bond strength tests conducted in our study, thus allowing for an external validity for a clinical scenario. In any case, this validity might be limited by the fact that the wire was applied as a small section on individual teeth, whereas for the in vivo case the same wire was bonded to more elements.

Considering that clinical studies should follow to confirm the in vitro results, a randomized clinical trial was conducted to test the same materials. The third null hypothesis was partially rejected. In both arches overall as well as in the upper and lower ones considered separately, significantly lower failure rates were assessed during a 2-year follow up for retainers bonded with the Scotchbond Universal adhesive after enamel pre-etching. Since the same orthodontic composite resin was used in this report, the two different bonding agents tested had a significant influence on the different number of failures assessed under clinical conditions. All the detachments assessed in vivo occurred at the enamel–adhesive interface, which appears as the most fragile (resin debonding); on the contrary, no detachment of the retainers from the adhesive (wire debonding) was disclosed. This is in accordance with a previous in vitro study, which showed that lingual appliances bonded on etched enamel almost left no adhesive after detachment; conversely, enamel pretreatment by means of sandblasting, before etching, resulted in more remnant adhesive on the tooth surface [42].

Within group 1, a significant difference between failures of the upper and lower arch was reported only for the lower teeth splinted with the traditional orthodontic primer which had significantly higher failure rates than the upper ones in the same condition (27.33% vs. 16.33%). Conversely, within group 2, a higher failure rate of the multistrand wires was assessed for the upper arch rather than for the lower one (7.33% vs. 6.00%), in accordance with other studies that attribute this outcome to occlusal factors [43]. However, this difference is not statistically significant in our study. Previous research, which considered a one-year follow up for retainers bonded with the conventional primer on the six lower anterior teeth, reported a 22.54% failure rate for multistrand stainless steel wires [9], compatible with the result here obtained. It is likely that the higher flexibility of the spiral wire absorbs energy itself, and the retainers bonding interface to enamel is less stressed by debonding forces.

The fourth null hypothesis was rejected. When comparing the clinical bonding failure on incisors and canines, the rates were significantly higher for the former, regardless of the adhesive and of the splinted arch. As reported in the review of Iliadi et al. [44], bonding retainers only to canines seem to avoid internal stresses of the wire, which occur in case of bonding to all six anterior teeth. It might be assumed that in this latter case, the multiple focal points of stresses prevail in the middle part of the wire which corresponds to incisors, whereas they are less pronounced at its extremities. This might explain the lower detachments assessed for canines, independently of the adhesive system considered. In any case, when using the universal adhesive in the total-etch mode, canines and incisors reported significantly lower failure differences between them than those assessed after the usage of the conventional primer, which suggests an influence of the bonding agent used. Since a different detachment rate depending on the dental elements was observed for both the mandibular and the maxillary arch, the different teeth surface existing when comparing the two anterior segment of the mouth seems not to have influenced this outcome.

Finally, the Kaplan–Meier survival curves clearly show a statistically higher survival rate for Scotchbond Universal (93.33%) following a 24-month follow up, rather than for the conventional Transbond XT Primer (78.17%). In any case, both these percentages are in accordance with the study of Tacken et al. [45], showing that the 2-year survival rate of multistrand retainers bonded with Excite^®^ (Vivadent, Schaan, Liechtenstein) was equal to 88%, considerably higher than the one reported by glass fiber reinforced bonded orthodontic retainers (49%).

The results obtained with the clinical trial disagree with those assessed in vitro but a direct comparison is not possible and, conversely, it would be misleading due to the different experimental conditions between the two studies. The rationale of the in vitro shear bond strength test is that of conducting an initial exploratory evaluation of the behavior of the two adhesives, limited to an ideal and short-term storage. A complete laboratory simulation is not always possible in dentistry because of the high variability occurring under in vivo conditions, which justify the need for having recourse to clinical trials [46]. In any case, it would be interesting to experiment other in vitro conditions such as a longer storage and a different storage medium (e.g., artificial saliva) or an artificial aging (by means of thermocycling or cyclic mechanical loading), which might disclose the different outcomes revealed in our clinical study.

Considering that both products tested in this study were applied on pre-etched enamel, the better outcome assessed in vivo for Schotchbond Universal should be ascribed to the chemical bond formation between 10-MDP and VP-copolymer of Schotchbond Universal with the apatite structure of acid-etched enamel that is missing from the Transbond XT Primer-etched enamel interface, the latter solely based on micromechanical retention.

The follow up here considered might be relatively short since patients should keep their bonded fixed retainer as long as possible in order to avoid orthodontic relapse. However, studies on this topic, as those considered in the systematic review of Al-Moghrabi et al. [47], generally report the survival rate over 12 to 24 months, and, to greater reason, retainers’ failures are more frequently observed during the first 6 months after their application [48].

It is well accepted that results of in-vitro studies might not replicate clinical applications [49,50]; as an example, bonding of the multistranded wire to only one tooth on the labial surface (unlike clinical situations where fixed retainer is bonded to the lingual surface of multiple adjacent teeth), the different enamel morphology, and tooth arrangement could also have attributed to the differences.

Future studies should be conducted to confirm the results here obtained and to verify whether the same outcomes referred to the adhesives here tested would be obtained even when bonding other type of wires, like fiber-reinforced composites retainers.

## 5. Conclusions

Despite the limitations of the two studies conducted, the universal adhesive Schotchbond Universal used in total etch bonding strategy could be a valid alternative to the conventional Transbond XT Primer for orthodontic retainers’ adhesion. Accordingly, the use of this product could be extended from restorative dentistry even to orthodontics.

## Figures and Tables

**Table 1:** Characteristics of the materials tested and application protocols recommended by the manufacturers.

Material	Type	Composition	pH	Application Protocol
Transbond XT Primer	Filler-free, light-cured liquid resin	BisGMA, TEGDMA	-	1. Teeth polishing with a non-fluoride oil-free pumice 2. Rinsing 3. Tooth isolation with cotton rolls 4. Etching 5. Thoroughly rinsing with water and thoroughly air-drying 1. Application of a thin uniform coat of Transbond XT Primer to each tooth surface to be bonded 2. Air-blow 3. Photopolymerization(10 seconds)
Scotchbond Universal	Universal adhesive	10-MDP, HEMA, silane, dimethacrylate resins, VitrebondTM copolymer, filler, ethanol, water, initiators, catalysts	2.7	According to the total-etch mode: 1. Tooth isolation2. Etching 3. Rinsing and air-drying 4. Adhesive application and rubbing for 20 seconds 5. Gently air-drying for 5 seconds 6. Photopolymerization for 10 seconds
Transbond XT Light Cure Adhesive Paste	Filler-reinforced, light-cured paste	Silane treated quartz (70%–80%), Bis-GMA (10%–20%), Bisphenol A Bis(2-hydroxyethyl ether) dimethacrylate (5%–10%), Silane treated silica (<2%), DPIHFP (<0.2%)	-	1. Apply around retainer 2. Photopolymerization for 20 seconds

Legend: bisGMA, Bisphenol A diglycidyl ether dimethacrylate; TEGDMA, Triethylene glycol dimethacrylate; 10-MDP, 10-Methacryloyloxydecyl dihydrogen phosphate; HEMA, 2-hydroxyethyl methacrylate; DPIHFP, Diphenyliodonium hexafluorophosphate.

**Table 2:** Shear bond strength (MPa) of the two products tested.

Group	Adhesive/Primer	Mean	SD	Minimum	Median	Maximum	Significance
1	Transbond XT Primer	11.05	2.43	8.39	10.51	18.63	*p >* 0.05; t = 1.948, df = 38
2	Scotchbond Universal	12.77	2.75	8.35	12.66	20.01	

SD: standard deviation.

**Table 3:** Frequency distribution of ARI indexes for each group. No significant difference was detected comparing groups 1 and 2 (*p* > 0.05; t = 2.067; df = 19), where both showed a higher frequency of ARI = 2.

Score	Group 1 (XT Primer)	Group 2 (SB)
ARI = 0	0	0
ARI = 1	5	10
ARI = 2	90	80
ARI = 3	5	10

ARI = adhesive remnant index.

**Table 4:** Numbers and rate of retainer failures for upper and lower teeth in the groups tested, with distinction between the type of teeth.

Group	Adhesive/Primer	Splinted Arch	Teeth	Teeth Bonded	Failures	Rate (%)	Significance
Group 1	Transbond XT Primer	Upper	Canines	100	7	7.00	*p* < 0.05
Group 1	Transbond XT Primer	Upper	Incisors	200	42	21.00	
Group 1	Transbond XT Primer	Lower	Canines	100	14	14.00	*p* < 0.05
Group 1	Transbond XT Primer	Lower	Incisors	200	68	34.00	
Group 2	Scotchbond Universal	Upper	Canines	100	3	3.00	*p* < 0.05
Group 2	Scotchbond Universal	Upper	Incisors	200	19	9.50	
Group 2	Scotchbond Universal	Lower	Canines	100	2	2.00	*p* < 0.05
Group 2	Scotchbond Universal	Lower	Incisors	200	16	8.00	

## Data Availability

Data are available upon reasonable request to Corresponding Authors.
